# Higher Adherence to Treatment With Low-Molecular-Weight-Heparin Nadroparin Than Enoxaparin Because of Side Effects in Cancer-Associated Venous Thromboembolism

**DOI:** 10.1097/HS9.0000000000000019

**Published:** 2018-02-02

**Authors:** Sake J. van der Wall, Frederikus A. Klok, Paul L. den Exter, Deisy Barrios, Raquel Morillo, Suzanne C. Cannegieter, David Jimenez, Menno V. Huisman

**Affiliations:** 1 Department of Thrombosis and Hemostasis, Leiden University Medical Centre, Leiden, The Netherlands; 2 Respiratory Department, Ramon y Cajal Hospital IRYCIS, Madrid, Spain; 3 Department of Clinical Epidemiology, Leiden University Medical Centre, Leiden, The Netherlands.

## Abstract

Current guidelines recommend low-molecular-weight-heparins (LMWH) monotherapy for 3 to 6 months as first-line treatment for cancer-associated venous thromboembolism (VTE). In clinical practice, enoxaparin and nadroparin are common agents used. However, differences in therapy adherence between these LMWHs have never been reported. Therefore, our aim was to compare adherence to enoxaparin and nadroparin in patients with cancer-associated VTE. Consecutive patients with active cancer and objectively confirmed VTE, treated at a Dutch or a Spanish hospital, were followed during LMWH therapy with a maximum of 180 days. Cumulative incidences of discontinuation of both LMWHs were estimated and compared according to the Kaplan-Meier method, applying a competing risk analysis to correct for mortality. A total of 366 patients were analyzed during LMWH treatment, of whom 284 patients (78%) were treated with enoxaparin and 82 (22%) with nadroparin. The cumulative incidence of discontinuation of enoxaparin and nadroparin treatment because of side effects was 30% (95% confidence interval [CI] 24–36) and 8.8% (95% CI 1.1–15), respectively. Competing risk analysis revealed a higher number of patients discontinuing enoxaparin due to side effects (adjusted hazard ratio [HR]: 2.8; 95% CI 1.06–7.2). Pain at the injection site was the most common reason of discontinuation in patients using enoxaparin, occurring in 32 patients, while it occurred in 1 patient using nadroparin (adjusted HR: 4.0; 95% CI 0.52–31). This analysis reveals that enoxaparin was associated with a higher risk of discontinuation because of side effects compared to nadroparin. However, given the nature of the patient groups, these findings should be followed by future studies.

## Introduction

Low-molecular-weight heparins (LMWH) are recommended for at least 3 to 6 months as first-line treatment for cancer-associated venous thromboembolism (VTE) by most current international guidelines because of proven superior efficacy compared with conventional vitamin K antagonists, with comparable risk of major bleeding.^1^,^2^,^3^

Recent research carried out at our institution has showed that 1 out of 5 patients with cancer-associated VTE stop LMWH injections because of side effects, mostly due to unacceptable pain at injection site.^4^ This finding was consistent with other smaller, retrospective studies reporting similar percentages of patients who switched to oral anticoagulants within 6 months.^5^,^6^ These studies, however, did not distinguish between LMWH preparations.

In clinical practice, enoxaparin and nadroparin are commonly used LMWH agents for treatment of (cancer-associated) VTE. These different LMWHs are prepared by a variety of chemical and enzymatic depolymerization techniques, resulting in marked differences in their physical and biochemical properties. These different characteristics might influence the burden of daily administration of subcutaneous injections. However, clinical data on the comparison of LMWHs is very limited and, so far, no single study has compared adherence with these LMWHs in patients with cancer-associated VTE. Two preliminary studies including heterogeneous patients have compared local tolerance of enoxaparin and nadroparin and suggested that the latter was locally better tolerated, possibly due to the difference in cationic salt composition.^7^,^8^ Thus, more accurate detailed information about adherence to different LMWHs for the treatment of cancer-associated VTE is required.

The aim of the current study was to compare adherence to daily subcutaneous injections of enoxaparin and nadroparin in patients with cancer-associated VTE.

## Materials and methods

### Study population

This was a prospective, multicenter, cohort follow-up study of consecutive patients with active cancer and objectively confirmed symptomatic proximal deep venous thrombosis (DVT) and/or pulmonary embolism (PE) to compare the adherence with enoxaparin and nadroparin during treatment with a maximum of 180 days. The design and characteristics of this cohort study have been described previously.^4^ However, in this study, only patients from the Leiden University Medical Centre (the Netherlands) and the Ramon Y Cajal hospital IRYCIS (Spain) with cancer-associated VTE between 2004 and 2014 and treated with therapeutic doses of LMWH were eligible for inclusion. In these hospitals, 2 specific LMWH preparations were used; in Spain, all patients were treated with enoxaparin (enoxaparinum sodium 100 mg [10,000 U/mL]) between 2004 and 2012 in the recommended dose of 1 mg/kg body weight twice daily (BID) in the first month, followed by as dosage of 1.5 mg/kg once daily (OD). In the Netherlands, all patients received weight-adjusted doses of subcutaneous nadroparin (nadroparinum calcium 9500 U/mL) between 2010 and 2014, either given once or twice daily—Fraxodi was given by 11,400 IU OD for patients under 70 kg and 15,200 IU OD for patients above 70 kg. Fraxiparine was given 5700 IU BID for patients under 70 kg and 7600 IU BID for patients 70 kg or more. At both hospitals, outpatient care comprised self-injections after standardized instructions by a trained nurse. All patients were followed during LMWH treatment with a maximum of 180 days and were excluded if they received other anticoagulants, were lost to follow-up or experienced a venous catheter-associated thrombosis.

The institutional review board of both the Leiden University Medical Centre and the Ramon y Cajal Hospital IRYCIS approved the study and waived the need for informed consent due to its observational design.

### Study endpoints

The primary objective of this study was to compare the discontinuation rate because of side effects of enoxaparin and nadroparin during the 6-month study period. Reasons for discontinuation were determined by the treating physician during hospital visitation and categorized as follows: local side effects defined as hematomas at injection side, site pain and exanthema, and heparin-induced thrombocytopenia. Patients were classified as having heparin-induced thrombocytopenia after a presumptive diagnosis, based on clinical parameters such as timing and degree of platelet count drop. The secondary objectives were to compare the incidences of recurrent VTE, major bleeding, and mortality of both LMWHs.

Recurrent lower extremity DVT was defined as new noncompressibility by ultrasonography of the common femoral and/or popliteal vein for lower extremity DVT in the transverse plane or the vein diameter under maximum compression, as measured in the abnormal venous segment, showing enlargement of thrombus diameter (>4 mm). Recurrent PE was defined as a new intraluminal filling defect on pulmonary angiography or computed tomography pulmonary angiography, a new high probability perfusion defect on V/Q scan or any new defects after earlier normalization of the scan, or confirmation of a new PE at autopsy. V/Q scans were evaluated according to the prospective investigation of pulmonary embolism diagnosis criteria. Major bleed was defined in accordance with the International Society of Thrombosis and Haemostasis criteria.^9^ Cause of death was verified by reviewing the pathology report. If autopsy was not performed, the likely cause of death was verified with the treating physician by reviewing the medical records and death certificates. All secondary outcomes were adjudicated within the study group.

### Statistical analyses

Means (standard deviation [SD]) and medians (interquartile range [IQR]) were used to present baseline continuous baseline variables for both LMWH groups. For categorical variables, we used frequencies and percentages. The Pearson's chi-square test was used to compare the distribution of the categorical variables, whereas the Mann-Whitney and independent *t* test were used for non-normal and normal distributed continuous variables respectively. For analysis of primary and secondary endpoints, follow-up started at the moment of first LMWH administration and ended at time of LMWH discontinuation or the maximum follow-up period of 180 days. The cumulative incidence of discontinuation of both LMWHs, recurrence VTE, and bleeding events were estimated according to the Kaplan-Meier method, presented with 2-sided 95% confidence intervals (CIs). A comparison was then made by a Cox-proportional hazard model, adjusted for gender, age, impaired kidney function and metastatic cancer, applying a competing risk analysis in which a patient was either censored for a specified outcome or not, and in the latter case completed the entire follow-up period (demonstrated with a hazard ratio (HR)).^10^ Data were analyzed using SPSS version 23 (SPSS Inc., Chicago, IL). A *P* value <0.05 was considered significant.

## Results

### Study population

A total of 366 patients were analyzed during LMWH treatment, of whom 284 patients (78%) were treated with enoxaparin and 82 (22%) with nadroparin (67 patients (82%) with Fraxodi OD and 15 (18%) with Fraxiparin BID). Table 1 shows the baseline characteristics of both LMWH therapies. Patients receiving enoxaparin were significantly older (mean age 68 years [SD 12] vs 62 years [SD 13]). Impaired kidney function and metastatic cancer were more present in patients treated with nadroparin (27% vs 9.7% and 63% vs 45%, respectively).

**Table 1:**
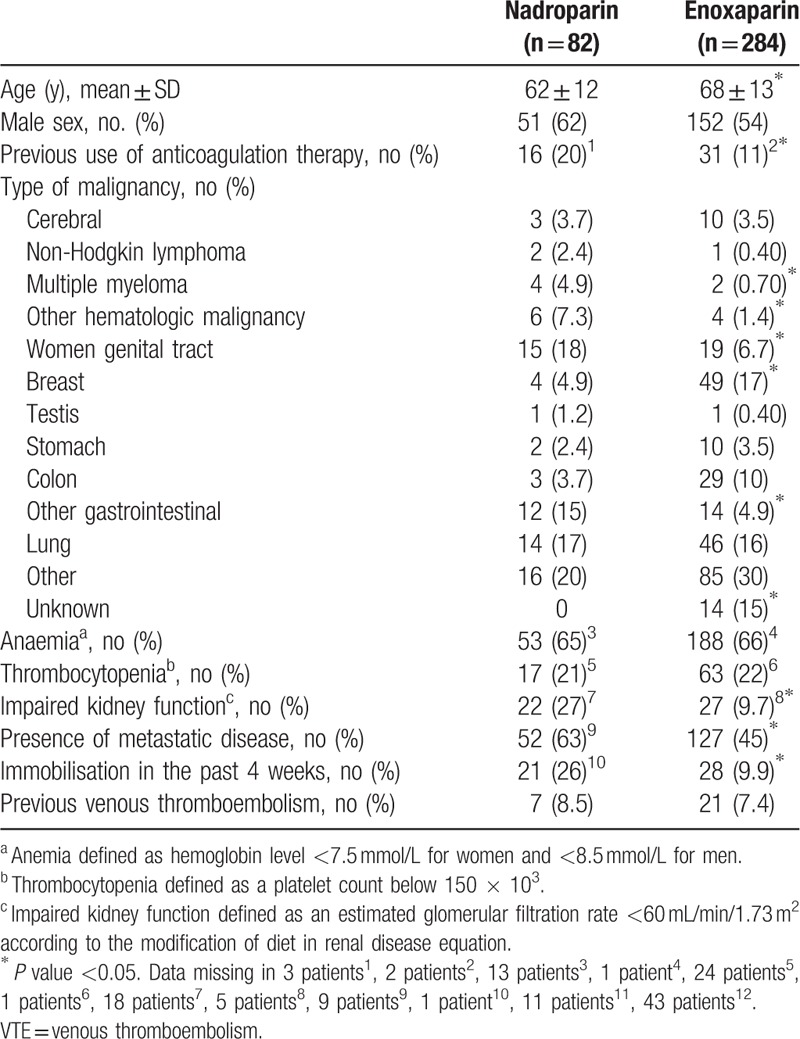
Baseline Characteristics of 366 Patients With Cancer-Associated VTE

### Discontinuation of LMWH treatment

**Figure 1:**
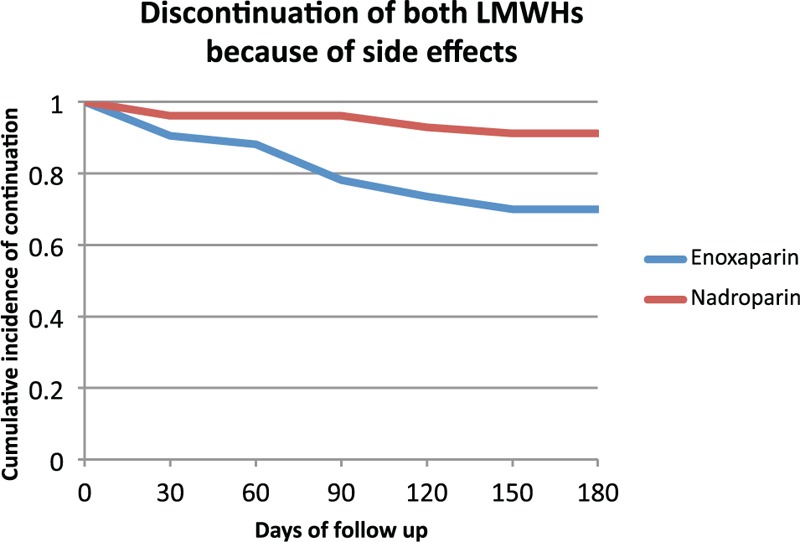
**Discontinuation of both LMWHs because of side effects after a maximum follow-up period of 180 days**. LMWH = low-molecular-weight-heparin.

Overall, 192 patients (52%) discontinued LMWH treatment within 6 months, of whom 151 patients (53%) were treated with enoxaparin and 41 patients (50%) with nadroparin. Reasons for discontinuation are shown in Table 2. A total of 77 patients (21%) discontinued LMWH treatment because of side effects, of whom 71 patients (92%) stopped enoxaparin after a median duration of 90 days (IQR 30–90 days) and 6 patients (7.8%) nadroparin after a median duration of 66 days (IQR 19–125 days; 5 patients using fraxodi OD, 1 patient using fraxiparin BID). The Kaplan-Meier survival for discontinuation of both LMWHs because of side effects is shown in Figure 1. The overall cumulative incidence of discontinuation during 6 months of enoxaparin and nadroparin treatment was 30% (95% CI 24–36) and 8.8% (95% CI 1.1–15), respectively. Competing risk analysis revealed a significant higher number of patients discontinuing enoxaparin (adjusted HR: 2.8; 95% CI 1.06–7.2).

**Table 2:**
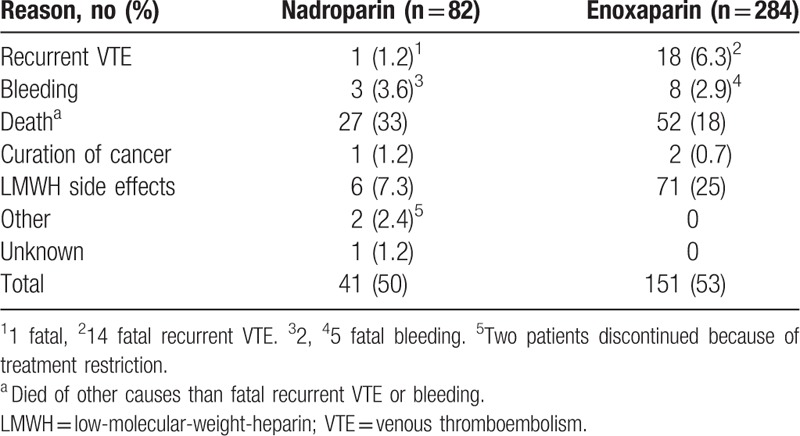
Reasons for LMWH Discontinuation

Table 3 shows LMWH side effects that led to discontinuation. Interestingly, pain at the injection site was the most common reason of discontinuation in patients using enoxaparin, occurring in 32 patients (cumulative incidence: 15% [95% CI 10–19]), while it occurred in 1 patient using nadroparin (cumulative incidence: 1.7% [95% CI 0–5.0]; adjusted HR: 4.0; 95% CI 0.52–31). Discontinuation because of local exanthema only occurred in 15 patients who were treated with enoxaparin (cumulative incidence: 7.1% [95% CI 3.6–11]).

**Table 3:**
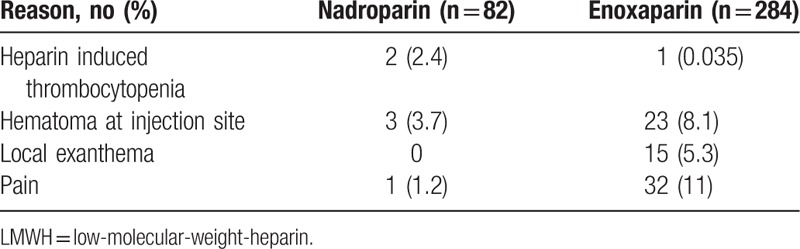
Reasons for LMWH Discontinuation Because of Side Effects

### Recurrent VTE, bleeding, and mortality

During the 6-month study period, a recurrent VTE occurred in 23 patients treated with enoxaparin after a median duration of 60 days (IQR 27–120 days) and in 6 patients treated with nadroparin after a median duration of 118 days (IQR 34–180 days), for a respective cumulative incidence of 11% (95% CI 6.4–15) and 7.6% (95% CI 1.8–13; adjusted HR: 2.9; 95% CI 0.65–13). Major bleeding events occurred in 27 patients using enoxaparin after a median duration of 90 days (IQR 30–120 days) and in 2 patients using nadroparin after a median duration of 66 days (IQR 34–66 days), for a respective 11% (95% CI 6.9–15) and 2.7% (95% CI 0–6.4) cumulative incidence (adjusted HR: 5.1; 95% CI 0.66–39).

Seventy-one patients died during enoxaparin treatment after a median duration of 60 days (IQR 30–90 days) and 30 patients died during nadroparin treatment after a median duration of 77 days (IQR 30–140), for a respective cumulative incidence of 29% (95% CI 23–34) and 39% (95% CI 29–49; adjusted HR: 1.3; 95% CI 0.76–2.3).

## Discussion

Our main observation was a significantly higher risk of discontinuation of LMWH treatment because of side effects of enoxaparin than of nadroparin in patients with cancer-associated VTE. During the 6-month study period, the adjusted hazard ratio of discontinuation because of side effects of enoxaparin was 2.8 compared with nadroparin treatment. These results elaborate on the findings of our previous study demonstrating a cumulative incidence of 1 out of 5 patients discontinuing both LMWHs due to side effects.^4^ The observed 30% cumulative incidence of discontinuation of enoxaparin was substantially higher than described in a previous study, reporting an incidence of 14% in a very small number of younger cancer patients treated with a similar dose.^11^ In comparison, the observed 8.8% cumulative incidence of discontinuation of nadroparin was consistent with those of a previous report, studying only patients with metastatic or locally advanced solid cancer.^12^

Pain at the injection site was the most common reason of discontinuation in patients using enoxaparin (45%), while occurring in only 1 patient using nadroparin (14%). This finding is in line with previous studies reporting a higher incidence of pain at the injection site in patients using enoxaparin than in patients using nadroparin, although these studies deal with different patient groups and a relatively short study period.^7^,^8^ They suggested that the pain intensity increased with the sodium concentration in enoxaparin, while in contrast, nadroparin is salified with calcium. Regarding pharmacodynamics and kinetics, only slight differences exist between both LMWHs.^13^,^14^,^15^ Thus, the sodium concentration in enoxaparin might be responsible for increased pain at the injection site, thereby leading to early discontinuation. However, since the proportion of salt dissolved in the LMWH preparations is almost negligible and other licensed LMWHs for the treatment of cancer-associated VTE (ie, tinzaparin and dalteparin) also contain sodium, this hypothesis seems unlikely. Unfortunately, no data were available on needle size differences of both LMWHs, which could also have contributed to our findings. A former study, however, found no reduction of pain and hematoma size in patients with cardiovascular disease using enoxaparin with 2 different needle gauges.^16^ Discontinuation because of local exanthema only occurred in 15 patients using enoxaparin (cumulative incidence: 7.1%). This finding differs from a previous prospective study demonstrating a higher incidence proportion of heparin-induced skin lesions in patients treated with nadroparin (17%) than enoxaparin (3.9%) in 321 patients who used LMWH for a minimum of 7 days.^17^ However, from all these reports, it is unclear whether the occurrence of side effects was a reason for discontinuation of therapy.

Comparative studies have not been performed to determine whether 1 LMWH is superior over the other in the treatment of cancer-associated VTE. In this study we found similar incidences of recurrent VTE and bleeding events of both LMWH agents.

This study has strengths and limitations. We included a large cohort providing novel and clinically relevant data on adherence to 2 different commonly used LMWH therapies in cancer-associated VTE. The most important limitation of this study was the non-randomized design. Both LMWHs were allocated according to the policy of the treating hospital and availability in the regional Dutch and Spanish pharmacies, thereby leading to differences in patient characteristics. Moreover, the evaluation of primary outcomes were not standardized, as treating physicians were only requested to report the reason of discontinuation and a HIT diagnosis was based on clinical assumption. For practical reasons, we combined 2 prospective databases (eg, Spanish and Dutch cohorts) with a different time frame of inclusion. We do not believe this would have influenced the discontinuation rate. During the 10-year inclusion period of enoxaparin, possible changes in composition or preparation techniques did not lead to different discontinuation rates. However, because of different inclusion durations, patients were not equally distributed among both groups. Additionally, in our adjusted analyses, it was not possible to correct for all potential confounders. Other characteristics such as social economic status and health coverage might also have influenced these findings. Furthermore, all Spanish patients were treated with enoxaparin injections BID for the first month, which could have led to a higher discontinuation rate. However, discontinuation of enoxaparin occurred only in 25% of the patients during the first month of BID administration. In comparison, of the 18% BID using nadroparin patients, only 1 discontinued during the 6-month treatment period. Hence, this was presumably of minor influence. Lastly, given the occurrence of relatively small number of individual reasons for discontinuation, our study did not achieve adequate power to detect possible significant differences between side effects of these 2 LMWHs.

In conclusion, our study reveals a significantly higher risk of discontinuation because of side effects of enoxaparin than nadroparin treatment in patients with cancer-associated VTE. However, these findings should be interpreted with caution owing to inherent patient groups, and more studies are needed to corroborate our findings.
